# Supplementary material for: Interaction Between Glucagon-like Peptide 1 and Its Analogs with Amyloid-β Peptide Affects Its Fibrillation and Cytotoxicity
Source: Int J Mol Sci. 2025 Apr 25;26(9):4095. doi: 10.3390/ijms26094095 (PMC12071944; doi:10.3390/ijms26094095)
Supplement: Supplementary file 1 [file ijms-26-04095-s001.zip › ijms-3578026-supplementary.pdf]

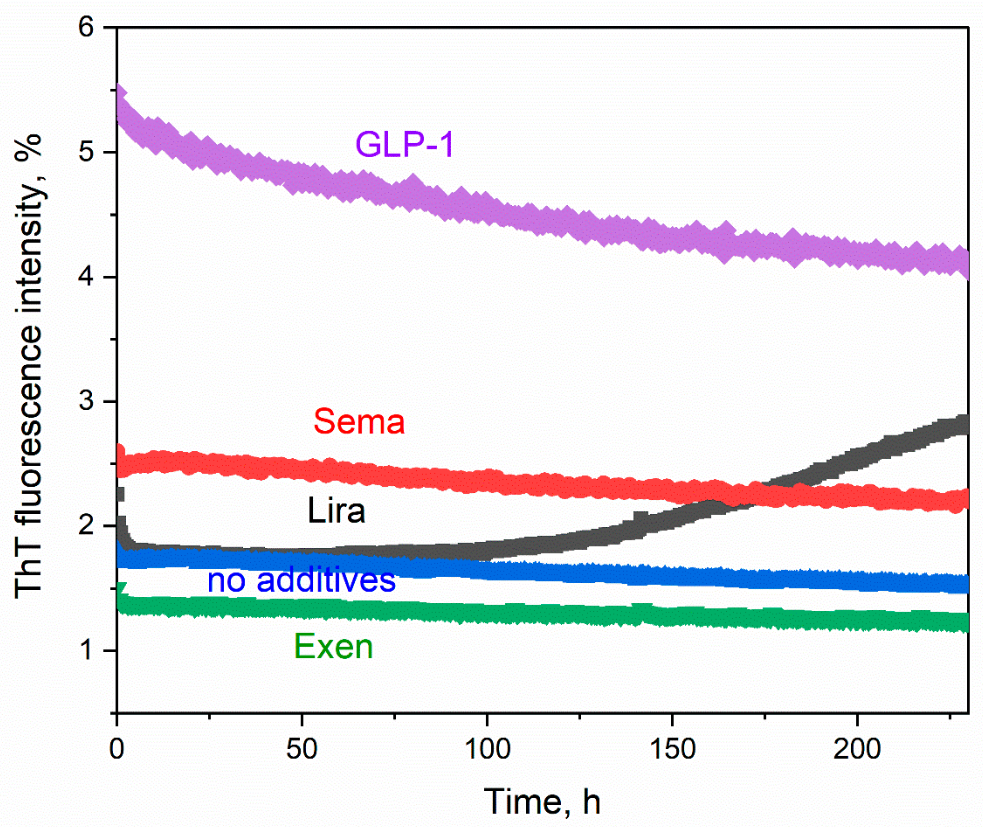

Figure S1. The change in ThT fluorescence in solutions with and without Sema (10 μM), Lira (10 μM), GLP-1 (10 μM), Exen (10 μM) over time. Buffer: 25 mM Tris-HCl, 140 mM NaCl, 4.9 mM KCl, 2.5 mM $CaCl_2$, 1 mM $MgCl_2$, 0.05% NaN_3, pH 7.4.
